# Characterization of a Spiking Convolutional Processor for FPGA

**DOI:** 10.3390/s26061801

**Published:** 2026-03-12

**Authors:** Dagnier A. Curra-Sosa, Francisco Gomez-Rodriguez, Alejandro Linares-Barranco

**Affiliations:** Neuromorphic Engineering Group of SCORE Excellence Unit (I3US), Department of Computer Architecture and Technology, EPS-ETSII, Universidad de Sevilla, 41004 Sevilla, Spain; gomezroz@us.es (F.G.-R.); alinares@atc.us.es (A.L.-B.)

**Keywords:** FPGA, DVS, Address-Event-Representation (AER), Spiking Convolution Neural Network (SCNN), LIF neuron model

## Abstract

In event-based neuromorphic processing, computer vision finds an efficient alternative capable of optimizing computational and energy resources, inspired by the dynamics of biological neural systems. In the development of real-time processing systems, it is crucial to visually represent the information captured by sensors and to explore its content with precision. Thus, machine learning models are implemented with the capability of being deployed on hardware devices with limited capabilities, depending on the intended purpose, ensuring savings in computational resources. The aim of this work was to evaluate the limits of the implemented neuron model, leaky-integrate and fire (LIF), for fitting convolutional layers of a neural network. To this end, the characteristics of the LIF neuron model used are summarized, as well as the details of its implementation in a hardware design, using configurable parameters. The experimental phase considered two convolution approaches to compare performance, Matlab R2022a software and a spiking convolutional processor for an FPGA, using sample recordings from the MNIST-DVS dataset and Sobel kernels for edge detection. The results reflect that the number of spikes generated by both approaches is very similar and their distribution by frame addresses is directly proportional.

## 1. Introduction

In recent years, neuromorphic engineering has gained relevance due to its ability to develop biologically inspired systems designed to solve complex tasks in real time. Its scope extends to various areas, such as sensory systems [1,2], robotics [3] and machine learning [4]. In this context, neuromorphic retinas stand out for their ability to identify patterns, edges, and other visual features in images, as well as to perform functions such as image recognition, object detection, and scene analysis. Thanks to their real-time visual processing, they are ideal for applications that require low latency and high speed, such as autonomous vehicles [5] and surveillance systems [2].

The Dynamic Vision Sensor (DVS) captures a scene in an innovative way, where each pixel acts like a neuron that detects changes in brightness and generates events in response to these variations. Unlike traditional frame-based cameras, which record the entire scene at fixed intervals, including pixels that have not changed, the DVS focuses solely on dynamic elements, omitting redundant information. Thanks to this approach, the amount of data to be processed is drastically reduced, since an event is generated only when a pixel detects a change in light, and it is transmitted immediately. This property enables asynchronous and continuous processing, eliminating the need for a fixed sampling rate and optimizing efficiency in computer vision applications and real-time response systems [6].

The rise of DVS sensors has driven the development of Spiking Neural Networks (SNN) and Spiking Convolutional Neural Networks (SCNN) designed to process data with temporal information, which traditional CNNs (Convolutional Neural Networks) cannot achieve efficiently. Thanks to their ability to handle the temporal dynamics of neurons, SCNNs make it possible to fully leverage the advantages of DVS, improving performance in tasks such as image classification and achieving greater energy efficiency [1].

One of its main advantages is pseudo-simultaneity [7], which allows processing to begin as soon as the first event from the sensor is received and enables real-time, uninterrupted results. However, this approach faces the challenge of high RAM consumption, since, unlike CNNs which only require memory for the computation of each layer, SCNNs must continuously store and update the membrane potential state of the entire network, without being able to apply resource multiplexing in memory. This is due to the fact that the arrival of any input event can trigger a chain reaction in the convolutional layers, potentially causing events to be emitted at any/several of them.

Although SCNNs offer significant advantages in processing dynamic visual information and temporal data, their implementation entails challenges in terms of training, optimization, and the development of specialized hardware. To address these obstacles, an interdisciplinary approach is required, combining advances in learning algorithms, optimization techniques, and efficient hardware architectures. The application of SCNNs in computer vision represents a paradigmatic shift in how systems perceive and process the environment, with key applications in action recognition [8], object tracking [9], anomaly detection [10], and event-based vision [11], the field to which this study belongs. These advances open up new possibilities for the development of autonomous systems and intelligent devices with efficient real-time processing.

In the literature reviewed, studies have been identified on SCNN implementations that consider the use of spiking datasets derived from DVS, as well as various learning and resource-saving strategies. In this regard, Cao et al. [12] demonstrate that their SCNN implementation on neuromorphic hardware is more efficient than its CNN equivalent in terms of energy. Vaila et al. [13] implement an SCNN with TensorFlow to study the behavior of the model according to learning parameters, as well as their effect on leakage and the weight initialization problem, training it with a variant of the STDP algorithm [14] and the MNIST and N-MNIST datasets [15]. Cordone et al. [16] trained a sparse SCNN with event-based data using PyTorch 2.6.0, demonstrating, through the performance achieved in terms of accuracy, sparsity, and training time with the DVS128 gesture dataset, the feasibility of using this bio-inspired approach for the future incorporation of real-time applications in low-power neuromorphic hardware.

The main contributions of this work can be summarized as follows:

- A hardware-oriented characterization of a spiking convolutional processor based on the LIF neuron model, implemented on an embedded FPGA platform and driven by real DVS data.
- A systematic analysis of the influence of LIF neuron parameters, including leakage, firing threshold, and refractory behavior, on output spike rates, event loss, and convolution under realistic event-driven workloads.
- A quantitative comparison between hardware-generated spiking outputs and a software-based convolutional reference, providing objective similarity metrics to validate the functional correctness of the spiking convolution implementation.
- An evaluation of architectural constraints such as memory buffering, FIFO saturation, and event throughput, highlighting practical limitations that arise when deploying SCNNs on resource-constrained neuromorphic hardware.
- Design guidelines and insights for the deployment of efficient, scalable SCNN layers on FPGA-based platforms, establishing a foundation for future multi-layer neuromorphic vision accelerators.

The structure of this article is organized as follows: Section 2 presents the background of the study, focusing on the particularities of the LIF neuron and its hardware implementation, while Section 3 describes the methodologies and experiments carried out to obtain a cost-effective combination of its configurable parameters. Section 4 explains the results derived from the experiments performed, as well as the evaluation of the obtained configuration by comparing it with a software-based approach. Finally, Section 5 presents the conclusions and future work directions.

## 2. Background

### 2.1. Dynamic Vision Sensor

Dynamic Vision Sensors, also referred to as event-based or neuromorphic vision sensors, represent a paradigm shift in visual acquisition, diverging from traditional frame-based imaging by outputting asynchronous events tied to changes in scene luminance. In contrast to conventional cameras that capture fixed-rate image frames regardless of motion, DVS pixels operate independently and emit an event only when there is a significant change in local brightness, yielding sparse spatio-temporal data with microsecond-level latency and extremely low power consumption [17]. This asynchronous signaling closely mimics biological retinas and has been shown to dramatically improve dynamic range, reduce motion blur, and minimize redundant data in scenes where temporal change is the key information—characteristics that have fueled considerable research interest and commercial adoption in recent years.

The first wave of DVS research established these sensors’ fundamental advantages and explored their potential in a variety of applications. For example, earlier work in event-based pedestrian detection used DVS leveraged event streams to detect and localize pedestrians more efficiently than frame-based counterparts, achieving significant improvement in detection accuracy and real-time processing speed on standard CPUs [18]. Similarly, research has demonstrated that DVS systems can be integrated into real-time force measurement and tactile sensing applications by capturing fine intensity changes and processing them with deep network architectures like LSTM-augmented convolutional models; this underscores the sensor’s versatility beyond traditional vision tasks [19]. Meanwhile, broader surveys and reviews of event-based vision support the idea that DVS technology is not merely a niche sensor variant but part of a broader neuromorphic ecosystem poised to transform computer vision, robotics, and embedded sensing with their high temporal resolution and efficiency advantages [20].

In parallel with sensor development, the most impactful advances in DVS research have arisen from processing architectures that exploit the native event-driven data format. Studies like that by Feng et al. propose a pure spiking architecture (DTEASN) tailored to the asynchrony, sparsity, and polarity attributes of DVS output, demonstrating improved performance metrics (latency, energy efficiency, and memory footprint) for object recognition and tracking tasks compared to conventional approaches [17]. These results underscore the potential of spiking backbones for near-sensor inference in embedded neuromorphic systems—a crucial step toward deploying DVS+SNN pipelines in edge devices.

Another important development lies in spatio-temporal feature extraction and attention mechanisms adapted to event streams. Works addressing trainable event-driven convolution with spiking attention show that extending standard event convolutions to learnable fixed kernels and attention modules can improve classification accuracy on benchmarks such as MNIST-DVS and CIFAR10-DVS, indicating that more sophisticated event representation and dynamic feature modeling enhance performance in classification tasks [21].

Complementing algorithmic advances, the review conducted by Akanbi & Ayomoh provides systematic perspectives on the promise and challenges of DVS integration in autonomous systems, surveying data processing bottlenecks, fusion strategies with other sensors, and calibration issues that persist in real-world use cases [22]. Such holistic treatments reinforce that DVS research is not limited to isolated classification milestones, but is ecosystem-level, spanning hardware optimization, spiking learning rules, benchmark datasets, and energy-aware inference strategies.

Compared to existing works in the literature, most DVS-based vision systems focus primarily on algorithmic accuracy or end-to-end application performance, often relying on software simulations or GPU-based implementations. Several studies demonstrate the advantages of combining DVS sensors with SNNs or SCNNs for classification, recognition, and tracking tasks, highlighting improvements in latency and energy efficiency (Table 1). However, these works typically abstract away the underlying hardware constraints, assuming ideal neuron behavior and unlimited memory resources.

In contrast, hardware-oriented approaches mainly emphasize architectural throughput or scalability, while offering limited insight into the detailed behavior of individual spiking neuron models under realistic event loads. As a result, the interaction between neuron-level parameters (e.g., leakage, refractory period, threshold) and system-level limitations such as memory bandwidth, FIFO saturation, and event loss remains insufficiently explored. The present work addresses this gap by providing an in-depth characterization of a LIF-based spiking convolutional processor implemented on FPGA, using real DVS data. By systematically exploring the configuration space of the LIF neuron parameters and comparing hardware-generated spiking outputs with software-based convolution results, this study bridges the gap between theoretical neuron models, software simulations, and practical hardware deployment of fully spike/event-based systems.

Early event-based convolutional architectures, such as those by Linares-Barranco et al. [23] and Camuñas-Mesa et al. [24], demonstrated the feasibility of spike-based convolution at the sensor interface but were limited either by reduced throughput, due to sequential processing, or by relatively high power consumption on FPGA solutions. Subsequent FPGA-based designs improved flexibility and scalability, as shown in [25,26], at the expense of increased power consumption or reduced throughput.

More recent spiking convolutional accelerators [27,28,29,30,31] mainly target frame-based input interfaces and hybrid processing pipelines, reporting performance in terms of frame rate, which prevents a direct comparison with event-driven throughput expressed in Mop/s. In contrast, the proposed architecture follows a fully event-driven I/O-core paradigm and reports sustained convolutional throughput independently of frame accumulation.

To better contextualize the proposed architecture, Table 2 provides a quantitative comparison with representative event-driven convolutional processors reported in the literature, highlighting their operating points in terms of throughput, latency, and power consumption.

Compared to previous event-driven designs, this work achieves a peak throughput of 348 Mop/s while supporting kernel sizes up to 7×7, exceeding the maximum kernel size of most FPGA-based spiking processors while maintaining the sub-milliwatt estimated power consumption (0.92 mW) of the core. This results in a significantly improved energy efficiency when compared to both early mixed-signal designs and recent high-performance FPGA accelerators, highlighting the suitability of the proposed architecture for low-power neuromorphic vision applications.

### 2.2. Leaky-Integrate and Fire Neuron Model

LIF neuron model is a simplified representation of the neuronal behavior used in computational neuroscience and in the development of SNNs. This model describes the dynamics of a neuron as a system that integrates input currents until it reaches an activation threshold, at which point it generates an electrical impulse or spike. During this accumulation, a leakage phase occurs, where the accumulated charge gradually dissipates if no new stimuli are received. Subsequently, once the spike is generated, the membrane potential resets and remains unchanged during the so-called refractory period.

Due to its balance between simplicity and ability to capture key aspects of neuronal activity, the LIF model has become a fundamental tool in the simulation of neuromorphic networks and in the study of information transmission in the brain. Over time, different researchers have worked on its characterization both in software simulations and in physical implementations using specialized hardware.

With regard to software, tools such as Brian [35] and NEST [36] have made it possible to simulate LIF neural networks with a high degree of flexibility. These simulators have been key to testing theories of neural processing, thanks to their ability to handle thousands or even millions of interconnected neurons. In particular, Brian stands out for offering an accessible environment for modeling neurons with customizable differential equations, while NEST has been used to validate large-scale hypotheses, such as the propagation of activity in cortical networks.

Other works, such as that of Gewers and Costa [37], present a transfer function-based approach to characterize the functionality of LIF neurons in terms of the instantaneous frequency of input and output signals, using successive numerical–computational simulations and statistical regressions. In the results obtained, they identify linearity in the transfer relationship, in conjunction with regions in the configurable parameter space.

In terms of hardware, one of the benchmarks is Intel’s Loihi chip [38], which implements LIF neurons in silicon to accelerate cognitive processes in real time. This chip enables the construction of neural networks that learn through local rules, such as time-dependent synaptic plasticity (STDP), and does so with very low energy consumption. Another important advance is IBM’s TrueNorth [39], which includes more than a million LIF-type digital neurons and has been used in applications such as visual pattern recognition. In addition, work such as those of Qiao et al. [40], Frenkel et al. [41], Moradi et al. [42], Richter et al. [43] and Linares et al. [44] have demonstrated implementations in FPGAs or ASICs that achieve a balance between biological accuracy and computational efficiency, allowing for greater adaptability in different applications. Some hardware convolutional processors based on LIF are available in the literature, such as those with event-based input/output: Ref. [45], which presented a VLSI analog implementation of a 16 × 16 pixels convolutional processor with a kernel of up to 16 × 16; Ref. [46], where a 64 × 64 convolutional processor is presented for kernels of up to 32 × 32 4-bit values processing the kernel pixel by pixel in a mixed-signal ASIC; and Ref. [25], where a generic convolutional processor is presented for FPGA with multikernel and row-updates capabilities. Furthermore, there are convolutional processors with a frame-based input/output but a spike-based processing core like in Refs. [26,27,29,30,31,32,33,47], which are quantitatively compared in Table 2.

These advances have made it possible to explore the potential of the LIF model beyond the academic field, bringing it closer to practical applications in artificial intelligence, autonomous robotics, and embedded systems, where speed and energy consumption are crucial.

### 2.3. Spiking LIF Formulae

To analyze the relationship between the input spiking frequency ($f_{\mathrm{in}}$) and the output spike frequency ($f_{\mathrm{out}}$) of a LIF neuron, at least the following parameters should be taken into account: the leakage constant ($\tau_{m}$), which represents the decay time of the membrane potential; the input current (*I*), which directly influences the increase in membrane potential; the firing threshold ($V_{\mathrm{th}}$), or the potential level required for the neuron to fire a spike; and the refractory time ($t_{\mathrm{ref}}$), which is the time that must elapse after an output spike before the neuron can fire again.

The mathematical model of the membrane dynamics of the LIF neuron [48] is described by Equation (1):

(1) $$\tau_{m}\frac{dV(t)}{dt}=-V\left(t\right)+R_{m}I\left(t\right)$$

where V(t) is the membrane potential, $R_{m}$ is the membrane resistance, and I(t) is the input current. The relationship between $f_{\mathrm{in}}$ and $f_{\mathrm{out}}$ is found by solving this system considering the key parameters.

The increase in potential due to an input event is proportional to the leakage constant and the current, ($\Delta V=\frac{R_{m}I}{f_{\mathrm{in}}}$). For the neuron to fire, the potential must reach the threshold $V_{\mathrm{th}}$. This implies that the time between spikes must satisfy $T_{\mathrm{spike}}=\tau_{m}\ln\left(\frac{V_{\mathrm{rest}}-V_{\mathrm{th}}}{V_{\mathrm{rest}}-V_{\mathrm{reset}}}\right)$, where $V_{\mathrm{reset}}$ is the potential to which it resets after a spike.

The output frequency is limited by $f_{\mathrm{out}}=\frac{1}{T_{\mathrm{spike}}+t_{\mathrm{ref}}}$. For low input frequencies ($f_{\mathrm{in}}$), the neuron does not fire for every input event, and $f_{\mathrm{out}}\propto f_{\mathrm{in}}$. For high input frequencies, the output frequency saturates due to the refractory time, such that $f_{\mathrm{out}}\approx \frac{1}{t_{\mathrm{ref}}}$.

For a transition zone, $f_{\mathrm{out}}$ increases nonlinearly with $f_{\mathrm{in}}$ due to the combination of the effects of $\tau_{m}$, $V_{\mathrm{th}}$, and $t_{\mathrm{ref}}$. Equation (2) captures the complete relationship between the input and output frequencies in a SLIF neuron:

(2) $$f_{\mathrm{out}}=\frac{1}{\tau_{m}\ln\left(\frac{V_{\mathrm{th}}-V_{\mathrm{reset}}}{V_{\mathrm{th}}-\frac{R_{m}I}{f_{\mathrm{in}}}}\right)+t_{\mathrm{ref}}}$$

**Figure 1:**
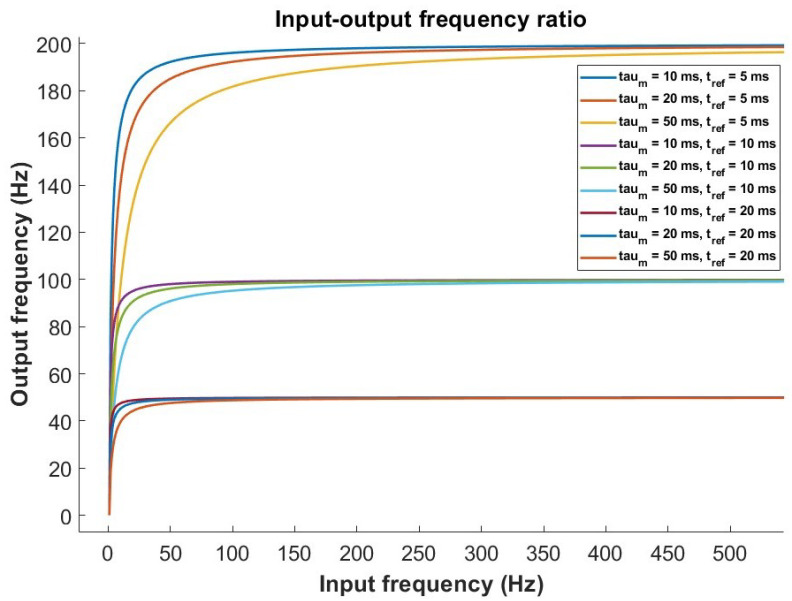
Theoretical behavior of the SLIF neuron model for various $\tau_{m}$ and $t_{\mathrm{ref}}$.

Figure 1 shows the behavior of the SLIF output spike frequency with respect to the input event frequency for different values of leakage constant and refractory time.

### 2.4. SCNN Accelerator

CNN models usually consist of three layers: the convolution layer; the pooling layer, to reduce its size and thus decrease the computation in future layers; and the nonlinearity layer, such as ReLU [49].

In frame-based image processing, the convolution operation is defined mathematically according to Equation (3), where *K* is the *N* × *M* convolution kernel, *I* is the input image, and *O* is the convolved image [50]:

(3) $$O\left(x,y\right)=\sum_{a=-\frac{N}{2}}^{\frac{N}{2}}\sum_{b=-\frac{M}{2}}^{\frac{M}{2}}K\left(a,b\right)\;\cdot \;I\left(a+x,b+y\right)$$

In contrast, in an event-driven processing framework, not every input pixel is processed, since neuromorphic sensors emit events only when there is a change in brightness in the observed scene. Each event is encoded as a tuple (x,y,p), where (x,y) denotes the coordinates of the pixel experiencing the change, and *p* is a polarity bit specifying whether the brightness variation is positive (ON) or negative (OFF) [51].

Assuming that the visual stimulus (events) from a DVS retina *I* is encoded so that each pixel I(x,y) is represented by a sequence of events, the outcomes of the partial convolution operations (computed for each received event) must be stored in a matrix *O* (implemented as capacitors in analog circuits or as registers/RAM cells in digital circuits). When an input event occurs, the corresponding pixel and its neighboring pixels are updated in *O* by adding the convolution kernel centered at the address of that event. Equation (4) formalizes this operation for an input event at (x,y):

(4) $$\begin{array}{cc} \; & O\left(x+a,y+b\right)=O\left(x+a,y+b\right)+K\left(a,b\right);\forall a,b\\ \; & a\;\epsilon \left[-\frac{N}{2},\frac{N}{2}\right],b\;\epsilon \left[-\frac{M}{2},\frac{M}{2}\right];N,M=dim\left(K\right) \end{array}$$

**Figure 2:**
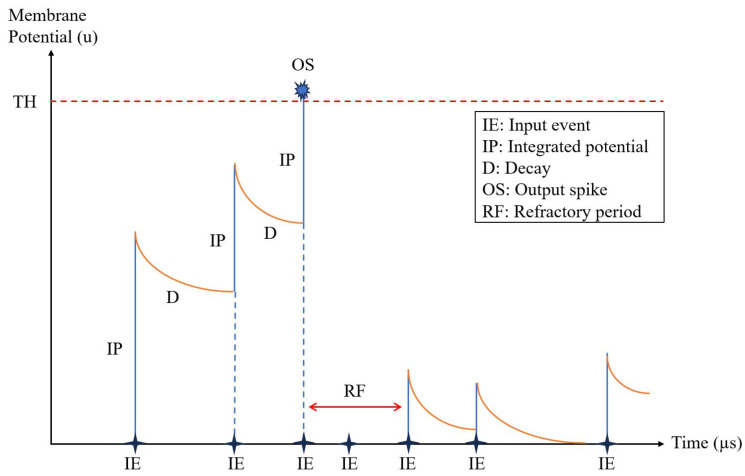
Changes in the membrane potential of a SLIF neuron over time due to input events integrations (blue) and neuron leakage (orange).

Once the events corresponding to pixel I(x,y) have been received and processed, the integrator associated with the address O(x,y) accumulates the contributions I(x+a,y+b)∀(a,b), each weighted by the kernel value, as defined in Equation (3). To avoid storing the convolution result in a full matrix of integrators *O*, this SCNN accelerator draws inspiration from the LIF neuron model [52]. In this model, the ongoing summation of kernel-weighted inputs to a neuron increases or decreases its membrane potential according to whether the coefficients are positive or negative. When a neuron’s membrane potential reaches a positive threshold (TH), it emits a spike with positive polarity at the address (x,y) and its membrane potential is reset, as illustrated in Figure 2.

A biological neuron decreases its membrane potential through leakage when it receives no excitation. The LIF neurons in the accelerator implement the leakage decay time, allowing the neuron to decrease its potential over time, thus controlling the rate of output events. Another quality of biological neurons is the refractory period. When a neuron fires and generates a spike, it must wait for a period of time before receiving any further excitation.

**Figure 3:**
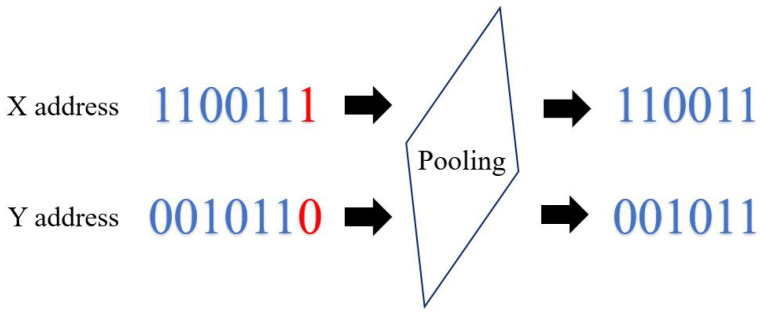
Example of event subsampling. The address values are divided by two with right shift.

In SCNN, subsampling (pooling) consists of dividing the (x,y) address of the output event of a convolution stage by two, reducing the size of the image (address space) [53]. In hardware, this step consists of shifting the x,y address one position to the right, as shown in Figure 3.

### 2.5. Convolutional Processor Architecture

The SCNN accelerator employed in this study is described in detail in [34]. It is a fully programmable, digital, event-driven convolutional system, inspired by the behavior of the LIF neuron. The architecture provides three interfaces: two AER interfaces for exchanging signals with neuromorphic systems, and a 32-bit digital interface that allows an embedded processor system to configure the accelerator. The system can perform up to 64 convolution operations in parallel, supporting kernel sizes ranging from 1 × 1 to 7 × 7. Input events are handled on a row-by-row basis, which lowers the latency per event relative to earlier implementations. The convolution engine also integrates the pooling operation.

To process a complete row, when an input event is received, each convolution engine retrieves from memory the membrane potentials, refractory timestamps, and leakage timestamps for the entire row. These parameters correspond to the neurons located around the event’s address, over the region defined by the programmed kernel size. The convolution engine then compares the timestamps against two global counters—one tracking the refractory period and the other tracking the leak—to determine whether the refractory period has elapsed and whether leakage should be applied. Next, the convolution engine combines a row of membrane potentials with a corresponding kernel row. Neurons that are allowed to fire and whose membrane potentials exceed the threshold generate an event at address (x,y). This procedure is repeated until every row has been convolved with all kernel rows.

### 2.6. Hardware Implementation

The design was specified at the RTL (Register–Transfer Level) using the SystemVerilog language, synthesized (default settings), and implemented (default settings) for a Zynq-7100 MMP platform with Vivado 2019.2 with a clock frequency of 100 MHz. This platform integrates a multiprocessor, reconfigurable MPSoC that employs a Dual-ARM Cortex-A9 as the processing system (PS) and a Kintex-7 FPGA as programmable logic (PL), providing 444 K logic cells on a single chip.

In this setup, the Zynq platform runs the Petalinux 2016.4 embedded operating system (OS) on the PS, enabling convenient system configuration for developers. The configuration is handled by a C++ program (available at https://github.com/RTC-research-group/SCNN_LIFrow (accessed on 22 February 2026)) that reads a text file containing the parameter values and transfers them to the PL, which interfaces with the PS through an AXI (Advanced eXtensible Interface). After the system has been configured, it transmits and receives events through the AER interfaces.

**Figure 4:**
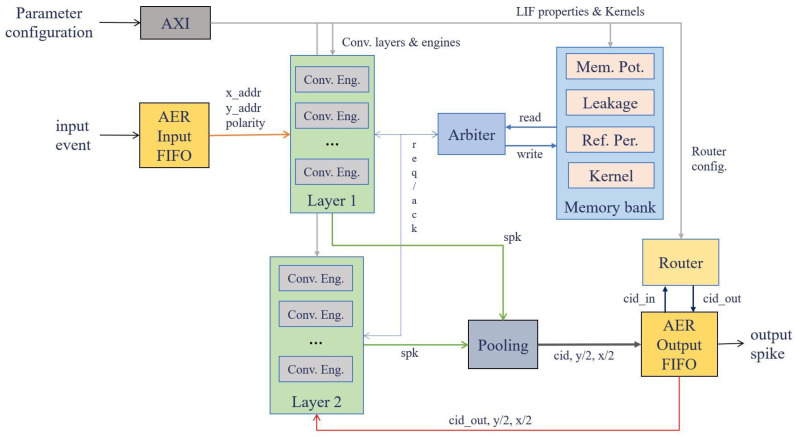
Block diagram of the system architecture.

The accelerator connects through two distinct bus types: an AXI slave bus [54], used to configure system parameters (leakage, decay, refractory period, threshold, kernel), and two AER buses [55,56], which handle incoming events from a sensor (or its recordings) and transmit output events for subsequent collection. The overall architecture is depicted in Figure 4.

Its workflow begins with an event entering the system, which is encoded in an AER format and stored in an input FIFO (up to 128 events). Next, the first-layer convolution engines take the event address and polarity in order to request read access from the memory arbiter to the banks that contain the values of the LIF neuron properties corresponding to the event’s pixel. At this point, the neuron state is checked to determine whether to apply leakage or refractory period, and then the counters for these properties and the membrane potential charge are updated. In addition, the kernel corresponding to the event polarity is accessed. Once these data have been received from the BRAMs, the convolution is performed, during which the defined threshold may be reached and a spike generated. The result of the convolution is updated by writing to the BRAMs, and the generated spike is sent to the output FIFO (up to 128 events) of the layer after its address is reduced through pooling. Here, the system router is consulted to decide, based on the spike’s CID, whether to send it to the second layer (when the CID belongs to the first layer) or outside the convolutional processor (when the CID belongs to the second layer). In the case of a spike that reaches the second layer, the same procedure as described for the first layer is applied.

The necessary resources for the FPGA (PL) part were approximately 212k LUTs (76%), 50k LUTRAMs (46%), approximately 170k flip-flops (30%), and 708 BRAMs (94%). The system requires an estimated dynamic power consumption (according to parameters shown in Table 3) of 573 mW for clocks, 639 mW for signals, 479mW for logic gates and LUTRAM, and 11mW for BRAM, adding up to a total of 1708mW dynamic power consumption for the FPGA.

### 2.7. MNIST-DVS Dataset

This dataset is available for free download (http://imse-cnm.csic.es/caviar/MNIST_DVS (accessed on 20 February 2026), provided it is used for non-commercial purposes and the original source is credited in publications and reports. It consists of a set of 30,000 DVS recordings with a resolution of 128 × 128 pixels, corresponding to the 10,000 samples from the original MNIST [57] at three different scales. These represent a slow saccadic movement of the digits projected from an LCD monitor, producing temporal contrast and DVS events at the edges of the digits.

**Figure 5:**
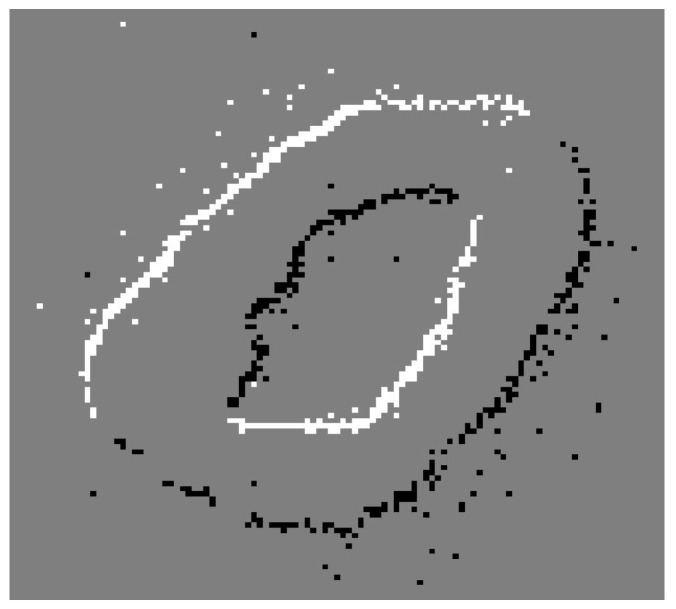
Sample recording histogram with ON (white) and OFF (black) events.

Its files with the extension “.aedat” can be played back using jAER software (v1.8.x) and edited using Matlab scripts designed to manage event information and generate new files with the same extension after they have been transformed. A static representation of the sample recordings can be seen in Figure 5, which shows an accumulation of positive (white pixels) and negative (black pixels) polarity events that delimit the outline of the digit during movement.

## 3. Methodology and Experimental Design

**Figure 6:**
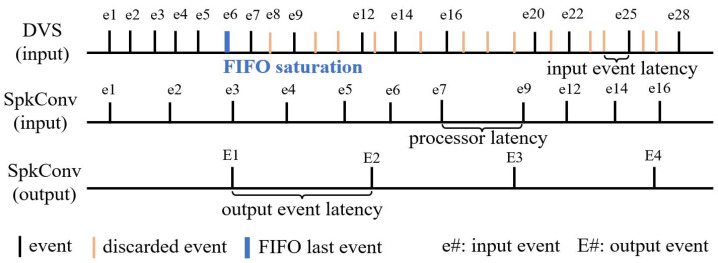
Example of an event flow.

Kernel application certainly entails selecting the elements to be processed, which leads to a loss of events in all scenarios. In this work, one of the objectives was to find a set of configurations that minimize the loss of events at the convolutional processor output once a kernel is applied. Among the situations that favor event loss, we considered the following three:

1. Input FIFO saturation: This occurs when the input event rate, whether from a DVS or from recorded and sequenced data, is higher than the operating throughput of the convolutional processor, which has a limit on the reception of events to be processed that depends mainly on the kernel size. Figure 6 depicts the problem. If the DVS event rate is faster than the convolution engine input rate, at some point, the input FIFO saturates, and input events will be ignored. The output event rate depends not only on the input rate, but also on the amount of input events required to produce an output event. This depends on many factors, such as the kernel size, values, decay, etc.
2. Convolutional processing: Convolution operation has several stages in which multiple computations are carried out, the system memory state is read and updated, and the components for event management and traffic are enabled. During processing, memory access the bottleneck occurs when the system arbiter allows only one convolution engine to read from and write to memory, in an amount determined by the kernel dimension, to perform the updates associated with the event being processed, a fact that introduces latency into the system. These tasks are repeated for each row of the convolution kernel for every event. Moreover, according to the implementation of the SLIF neuron, if the increase in membrane potential from the kernel is high and the leak rate is small, its refractory period will be triggered more frequently. This property is sensitive with respect to event loss when its value is higher than the average event rate per address.
3. Output FIFO saturation: This occurs when the output event rate of the convolution engine is higher than the capacity of the external system that receives its events, in our case, a usbaermini2 [55] with a maximum supported monitoring rate of 5 Mevps.

In the experiments carried out, the previously described neuromorphic MNIST-DVS dataset was used. The set of events that occur during the playback of the dataset files are sequenced by an usbaermini2 board towards the convolutional processor. Its output, represented by spikes generated by the convolution engines that compose it, is monitored by a second usbaermini2 board to maximize its input event rate. This output, with a format similar to the input, is the one on which we carry out various studies to characterize the behavior of the convolutional processor, so as to enable decision-making with a view to the future deployment of an SCNN model.

### 3.1. Configuration Parameters

As described in Section 2.3, Threshold is the maximum charge to be reached by the SLIF neuron membrane potential to emit a spike, while Leakage represents the decay rate of the membrane potential over time and the Refractoryperiod is the mandatory inactive time of the neuron after firing a spike. The different properties of the LIF neuron are represented in hardware by descriptive parameters that store its state, synchronized with the system’s main counter. The configuration of the convolutional processor deployed on the FPGA is provided from the embedded ARM computer, using an application developed in C, which decodes such configuration from a text file generated in Python 3.9.16 (parser). This file’s line-by-line content (in hexadecimal) is as follows:

1. Convolution engines ID (1 to 64);
2. Convolution engines layer distribution;
3. Threshold (8 ubits);
4. Leakage;
5. Kernel dimension;
6. Refractory period;
7. Prescalers for leakage and refractory period;
8. Kernel values.

The leakage property (theoretical $\tau_{m}$) comprises two parameters: value and decay, which simulate the leakage process of the neurons’ membrane potential. The parameter value (7 ubits) is the number of leakage-counter updates. After this time, a decrement equal to decay (8 ubits) is applied to the membrane potential. In the same way, the refractory period ($t_{ref}$, 7 ubits) indicates the number of updates of the refractory period counter to determine the rest time for the LIF neuron once it has fired, during which it is not allowed to emit a spike.

**Figure 7:**
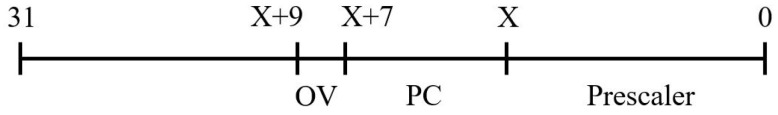
Property counters derived from prescaler and system counter (OV: overflow; PC: property counter).

The prescalers (5 ubits) establish the number of LSB bits from a counter to be skipped for leakage and refractory period calculations (Figure 7). We take as a reference the main counter of the circuit (32-bit) according to the configured clock frequency (in this case 100 MHz). Therefore, their values represent the number of bits shifted to the left in the main counter, from which the needed bits for these properties are derived. Finally, the kernel values are encoded one per line together with their position in the representative matrix.

For the characterization of the convolutional processor, the starting point was the configuration used in [34]. In that work, in broad terms, the validity of implementing a multi-convolutional system on FPGA was verified, ensuring parallel processing (by rows) with low latency in data transfer. The values for the parameters described above are proposed in Table 4.

From these values, for leakage and refractory period properties (p={lk,rp}), it is possible to obtain the time ($T_{p}$) for the leakage and refractory period properties by considering the values ($V_{p}$) of the bit windows established by the respective prescalers ($P_{p}$) and the clock frequency ($F_{c}$) at which the convolutional processor operates, according to Formulation (5):

(5) $$T_{p}=\frac{V_{p}\;\cdot \;2^{P_{p}}}{F_{c}}$$

Thus, as the leakage time, the decay of the membrane potential occurs after $80\;\cdot \;(2^{10}\;\cdot \;10)$ ns, that is, 819.2 μs. Meanwhile, the neuron’s downtime, once it enters the refractory period, would be $50\;\cdot \;(2^{8}\;\cdot \;10)$ ns, equivalent to 128 μs. Moreover, the samples were processed with Sobel kernels to determine vertical and horizontal edges, achieving latencies below 10 μs. However, no prior study has determined the optimal configuration to mitigate event loss during processing and to keep the input and output event rates in a proportional relationship before the saturation of output frequency, as suggested by theoretical models of the LIF neuron.

### 3.2. Validation of the Spiking Approach

With the aim of minimizing event loss, we proposed several experiments to find a better combination of values for the configuration parameters. Therefore, due to the large number of possible configuration options, the first part consisted in an exploration using constrained samples that contained only a single neuron and an inter-event interval fixed to the average processing time of the convolutional processor.

Once a stable configuration was determined for the possible combinations of the convolutional processor parameters, an experiment performing the convolution with Sobel kernels was carried out. At this point, two approaches were considered: the first approach (software) using Matlab’s conv2 function and the second approach (hardware) using the convolutional processor.

Although the proposed convolutional processor operates using spiking leaky-integrate and fire (SLIF) neurons with thresholding, leakage, and refractory dynamics, its output can be meaningfully compared to a classical linear convolution under a specific operating regime. In particular, when the input event rate is sufficiently high and the membrane time constant $\tau_{m}$ is large relative to the inter-event interval, the membrane potential effectively integrates incoming events over a temporal window, behaving as a leaky accumulator. In this regime, the contribution of individual events to the membrane potential is approximately linear, and the leakage term mainly determines the temporal extent of integration rather than dominating the dynamics.

Furthermore, when the firing threshold is set such that multiple events are required to elicit a spike, the emitted spike count over a fixed observation window becomes proportional to the accumulated membrane potential prior to thresholding and reset. As a result, the spike histogram at the output of the SLIF neuron approximates a scaled and quantized version of the linearly accumulated input. When extended to a convolutional layer, this mechanism effectively implements a spatio-temporal filtering operation whose steady-state response corresponds to the convolution of input event histograms with the kernel weights. Under these conditions, the spike-based output preserves the spatial structure of the linear convolution, enabling a meaningful comparison between the hardware-generated spike histograms and the software-based convolution of accumulated event frames.

**Figure 8:**
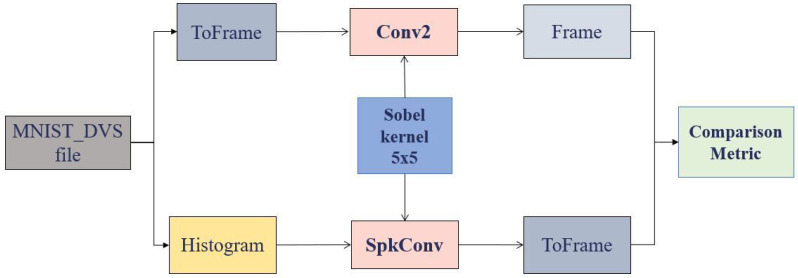
Computation of the similarity between convolution operations.

To compare software and hardware approaches, histograms with 500 events were extracted from the sample recordings and transformed according to the flow shown in Figure 8. These histograms were selected at the beginning (H1), the middle (H2), and the end (H3) of the .aedat file. To process their content, they were transformed into frames through the accumulation of events, in order to ensure that the metrics can evaluate their similarity based on their representative matrices.

In this experiment, variability was also added to the threshold values and the Sobel kernel (Equation (6)) itself by including an increment scale. According to the considered threshold range, 11 possibilities were evaluated in increments of five units, while the scale to be multiplied by the kernel values was taken from 1 to 10. The histograms of events obtained for each combination were compared with their counterparts from the Matlab function, for which the pairs with the greatest stability and best metric values were identified.

(6) $$S_{h}=\left[\begin{array}{ccccc} 2 & 2 & 4 & 2 & 2\\ 1 & 1 & 2 & 1 & 1\\ 0 & 0 & 0 & 0 & 0\\ -1 & -1 & -2 & -1 & -1\\ -2 & -2 & -4 & -2 & -2 \end{array}\right],\;S_{v}=\left[\begin{array}{ccccc} 2 & 1 & 0 & -1 & -2\\ 2 & 1 & 0 & -1 & -2\\ 4 & 2 & 0 & -2 & -4\\ 2 & 1 & 0 & -1 & -2\\ 2 & 1 & 0 & -1 & -2 \end{array}\right]$$

To quantitatively assess the similarity between hardware- and software-generated representations of the Sobel convolutions, a set of complementary metrics was employed, capturing both point-wise agreement and global distributional consistency. Firstly, a binary coincidence ratio (BCR) was computed by comparing binarized event maps, where each pixel is assigned a value of one if at least one event is present and zero otherwise (Equations (7) and (8)). This metric measures the proportion of spatial locations exhibiting identical activity, providing an intuitive estimate of overall agreement [58,59].

(7) $$F_{SW_{b}}\left(i,j\right)=\left\{\begin{array}{cc} 1, & F_{SW}\left(i,j\right)>0,\\ 0, & \mathrm{otherwise}, \end{array}\right.\;F_{HW_{b}}\left(i,j\right)=\left\{\begin{array}{cc} 1, & F_{HW}\left(i,j\right)>0,\;i,j\in \left[1,N\right]\\ 0, & \mathrm{otherwise}. \end{array}\right.$$

(8) $$BCR=\frac{1}{N^{2}}\sum_{i=1}^{N}\sum_{j=1}^{N}\left(F_{SW_{b}}\left(i,j\right)==F_{HW_{b}}\left(i,j\right)\right),$$

Here we have $F_{SW}$ and $F_{HW}$ as the software- and hardware-generated frames, respectively, with $F_{SW_{b}}$ and $F_{HW_{b}}$ as their binarization and *N* as the matrix dimension.

To further characterize differences in event magnitude and spatial distribution, magnitude-based and distribution-based metrics were also considered. The Root Mean Squared Error (RMSE) was computed between corresponding event accumulation maps to capture local differences in activation strength, complementing the binary similarity measures with a magnitude-sensitive criterion [60]:

(9) $$\mathrm{RMSE}=\frac{1}{N}\sqrt{\sum_{i=1}^{N}\sum_{j=1}^{N}{\left(F_{SW}\left(i,j\right)-F_{HW}\left(i,j\right)\right)}^{2}}$$

Moreover, the Kullback–Leibler (KL) divergence was employed to evaluate the dissimilarity between normalized spatial event distributions, interpreted as probability mass functions (Equations (10) and (11)). Since KL divergence is asymmetric and sensitive to zero-probability events, the Jensen–Shannon (JS) divergence was additionally reported as a symmetric and numerically stable alternative. The JS divergence (Equation (12)) provides a bounded measure of distributional similarity and has been widely used in information-theoretic analyses of neural and event-based data [61].

(10) $$P\left(i,j\right)=\frac{F_{SW}\left(i,j\right)}{\sum_{i,j}F_{SW}\left(i,j\right)},\;Q\left(i,j\right)=\frac{F_{HW}\left(i,j\right)}{\sum_{i,j}F_{HW}\left(i,j\right)},\;M=\frac{1}{2}\left(P+Q\right)$$

(11) $$D_{\mathrm{KL}}\left(P\|Q\right)=\sum_{i,j}P\left(i,j\right)\log\frac{P(i,j)}{Q(i,j)}.$$

(12) $$D_{\mathrm{JS}}\left(P,Q\right)=\frac{1}{2}D_{\mathrm{KL}}\left(P\|M\right)+\frac{1}{2}D_{\mathrm{KL}}\left(Q\|M\right),$$

In these formulations, *P* and *Q* represent the normalized distributions of events, $D_{KL}$ is the Kullback–Leibler divergence, and $D_{JS}$ is the Jensen–Shannon divergence. Together, these metrics enable a comprehensive evaluation of both local correspondence and global structural consistency between hardware and software event representations.

## 4. Results and Discussion

The test setup consisted of two USBAEARmini2 interfaces for sequencing events to the SCNN processor and to monitor events from the SCNN processor. These USB interfaces have a maximum sustained throughput of 5 Mevps [55], and the SCNN processor has a sustained throughput from 0.77 Mevps to 0.11 Mevps for kernel sizes from 1 × 1 to 7 × 7, respectively [34].

**Figure 9:**
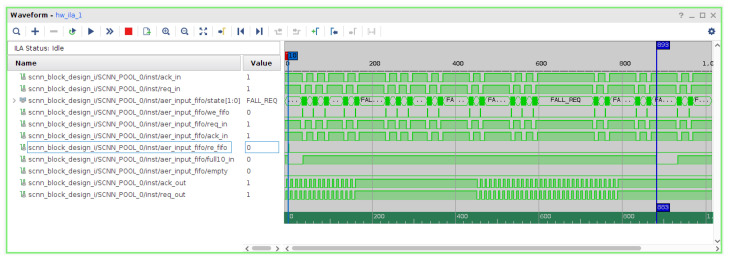
Temporal window for FIFO state during processing.

To determine these event transfer rates in the FIFOs, as well as their saturation levels, a test was conducted to capture the platform’s performance during the processing of a sample recording. Kernels of dimension 1 × 1 and 7 × 7, corresponding to the minimum and maximum possible values for our architecture, were used to obtain the maximum and minimum rates, respectively. The measurements yielded a latency of 130 clock cycles for the 1x1 kernel and 893 for the 7 × 7 kernel (Figure 9). Additionally, the experiment showed an output event latency of 11 clock cycles, equivalent to a rate of 9.09 Mev/s.

Figure 9 shows an excerpt of the internal AER input FIFO signals captured using the Vivado Integrated Logic Analyzer (ILA) under sustained high event-rate stimulation. Although the FIFO does not reach its full condition (full10_in=0), the trace reveals periods where write operations are temporarily stalled (we_fifo=0) due to downstream backpressure. During these intervals, the AER interface remains in the FALL_REQ state, indicating that incoming event requests cannot be acknowledged at the maximum input rate. This behavior demonstrates a throughput-limited operating regime in which the event producer attempts to inject data faster than the spiking convolutional core can process it. Importantly, no event loss due to FIFO overflow is observed, confirming that the proposed architecture enforces flow control at the interface level and degrades gracefully under high event loads. These measurements highlight that the effective saturation point of the system is determined by the internal processing latency of the spiking convolutional pipeline rather than by FIFO capacity constraints, providing a realistic characterization of the platform’s sustained event-handling capability.

To evaluate the system behavior under different parameter configurations, a test was conducted to identify the settings that minimize event loss. The kernel used consisted of a impulse (Dirac-like) 5 × 5 kernel which had an identical value (equivalent to $R_{m}I$ in Equation (2)) to the proposed threshold in the central position and zero in the remaining ones. The processed samples consisted of examples of each of the digits taken from the dataset.

For the initial experiment, the six parameters were separated into two groups. The first one consisted of the following parameters: threshold, leakage decay, and values for leakage and refractory period and the second with both prescalers. Table 5 shows the values used in the 300 different tests carried out corresponding to the combination of different values for the parameters in group 1. In the case of prescalers (group 2), the step size was 1 for a total of 441 values per combination of group 1 parameters. In all cases, the output events generated by the convolution system were obtained by processing five runs for each of the five samples per digit.

**Figure 10:**
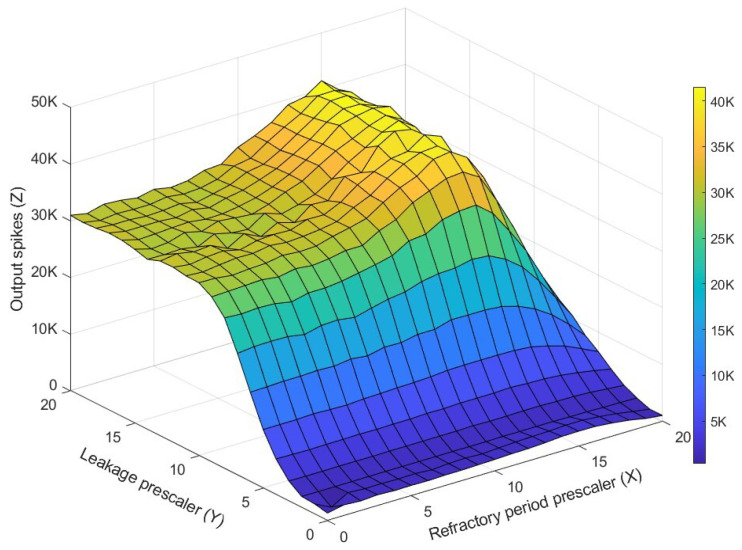
Output spikes for each combination of prescaler values and the remaining fixed parameters (threshold = 50, decay = 0, leakage value = 80, refractory period value = 1).

After conducting tests with every possible combination of parameters, the pairs of prescaler values that produced the 10 highest values for the output spikes were collected per run. A trend in the appearance of value ranges was identified, from which the most stable pair in most of the tests performed was selected. These values were: 19 for the leakage prescaler and 14 for the refractory period prescaler. As an example of the graphs generated in these tests, Figure 10 shows the best case shape. This shape was the same for similar good cases.

As can be observed in the Figure 10, for small values of the prescalers, whether for the leakage or the refractory period, there are not many output spikes generated compared to those received. This is mainly due to the constant overflow of the counters for these properties, which means that the convolutional processor must spend more time updating the LUTRAMs, which take 2.56 μs per row [34]. As the leakage prescaler increases, the output spikes increase until they stabilize once the prescaling reaches 10 bits. Similarly, when looking at the axis representing the refractory period prescaler, the output spikes show constant behavior for values below 10 bits, before reaching the optimal range that maximizes the value of this function.

With these prescaler values, a study was conducted to obtain an initial approximation of the event filtering limit for the convolutional processor. Table 6 also shows the improvement in terms of reducing lost events compared to the initial configuration (Table 4 prescalers).

In this case, a sample was taken per digit for which the input throughput shown in the second column was determined. The next two columns show the output throughput and the associated loss with respect to the input throughput for the initial configuration, which were calculated from the average of three runs with ten trials per digit sample. Similarly, the last two columns represents the results obtained for the prescaler values that minimize event loss, showing a significant improvement in the reduction in events lost from around 49% to 18%.

In the following experiment, standard values were set for the properties of the LIF neuron in order to verify, with respect to its theoretical model, the behavior of the SLIF neuron implemented in the convolutional processor. The leakage time and refractory period adopted were established using Formula (5), based on the previously obtained prescalers.

**Figure 11:**
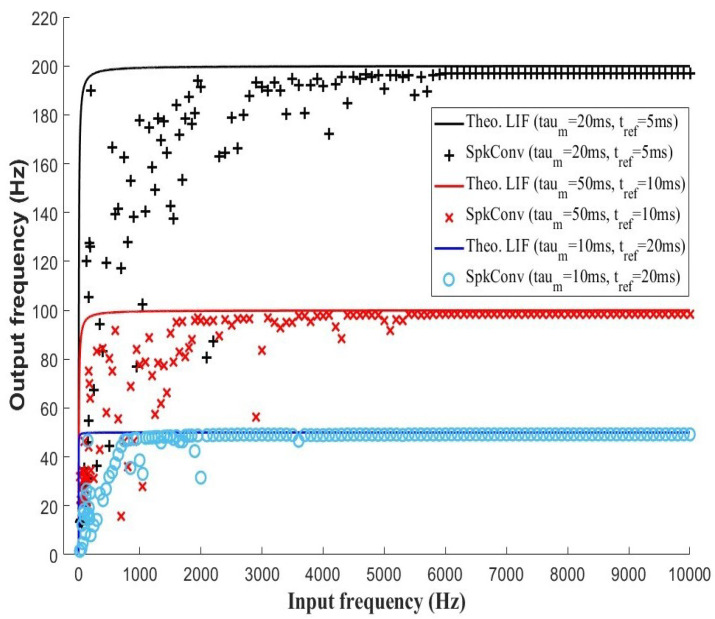
Relationship between output frequencies for theoretical and empirical LIF.

Figure 11 illustrates the relationship between input and output firing rates for different combinations of the membrane time constant $\tau_{m}$ and the refractory period $t_{\mathrm{ref}}$, comparing experimental measurements obtained from the convolutional processor with the theoretical LIF model. In all cases, the input frequency was taken as the inverse of the inter-spike interval, which varied in value from 500 μs to 10 ms while the output frequency exhibits the characteristic nonlinear behavior predicted by the model: a rapid increase at low input rates followed by saturation at higher frequencies.

This saturation is primarily governed by the refractory period, which imposes an upper bound on the maximum achievable firing rate. As observed experimentally, increasing $t_{\mathrm{ref}}$ shifts the saturation plateau toward lower frequencies, while smaller values allow the neuron to sustain higher output rates, in agreement with the theoretical expression.

The membrane time constant $\tau_{m}$ mainly affects the slope of the response curve in the sub-saturation regime. Larger values of $\tau_{m}$ lead to a slower accumulation of membrane potential, resulting in a reduced sensitivity to input frequency variations at low and intermediate rates. Conversely, smaller $\tau_{m}$ values produce a steeper initial slope, enabling faster integration of incoming events. While the theoretical model predicts an idealized continuous response, the experimental curves display noticeable dispersion and a slight reduction in the maximum firing rate. These deviations are attributed to system-level effects such as discrete-time implementation, event arbitration, buffering, and communication latencies, which effectively increase the refractory interval. Nevertheless, the overall trends observed across all parameter configurations closely follow the theoretical predictions, confirming that the convolutional processor preserves the expected LIF dynamics and that $\tau_{m}$ and $t_{\mathrm{ref}}$ retain their functional roles in shaping the firing-rate response.

Next, a comparison between the convolution operations (software vs. hardware) was performed to determine the similarity between both approach outputs. This test was based on convolute Sobel kernels over the three histograms (H1, H2, and H3) for all the pairs of threshold and kernel_scale considered, and the metric value ranges are summarized in Table 7.

These results show a consistent agreement between the software-based convolution and the event-driven convolutional processor across all evaluated sections and Sobel kernels. High values of the BCR, exceeding 0.92 in all cases and reaching up to 0.97, indicate a strong correspondence in the spatial support of the most significant responses, leading to a closer alignment between the frame-based and spiking representations.

In contrast, point-wise error metrics such as RMSE exhibit limited discriminative capability in this context, remaining relatively constant across sections and kernels despite the clear visual similarity between the resulting convolution frames. This behavior is expected given the sparse, non-negative nature of the event-based output compared to the dense, bipolar response produced by conventional convolution. Moreover, the Jensen–Shannon divergence yielded consistently low values, particularly for the vertical Sobel kernel, confirming a high similarity between the output distributions of both approaches. Taken together, these results demonstrate that while traditional error metrics may underestimate the correspondence between standard and neuromorphic convolutions, structure- and distribution-aware measures provide a more meaningful and reliable assessment.

**Figure 12:**
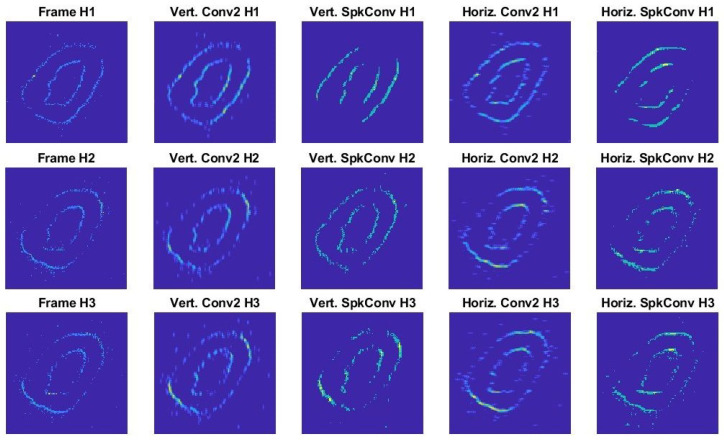
Frames generated by Sobel kernels.

This fact is noticeable due to the color density of the compared frames in Figure 12, which were generated with the following parameters: threshold=50, decay=0, refractory_period_time=0.164μs (derived by refractory_period_value=1 and refractory_period_prescaler=14 in Equation (5)) and kernel_scale=1.

In the figure, the first column (from left to right) corresponds to the frame processed. The second and third columns show the output generated by Matlab’s conv2 function, and the last two show the convolutional processor output. As for the rows, in order, they represent the three histograms studied from the sample recording. For both approaches, there was an approximate decrease in activity in the transition through the histograms from H1 to H3. However, for the histograms generated by the convolutional processor, the difference was minimal compared to the kernel applied, in contrast to what can be seen for the Matlab outputs. This confirms that the proposed event-driven convolutional processor effectively preserves the essential structural information of classical convolution kernels despite its inherently asynchronous and sparse operational paradigm.

This analysis shows the influence of the convolutional processor configuration on the values of the parameters representing the properties of the implemented SLIF model. The study of its specific design clarifies the reason for the significant loss of spikes generated compared to those received, providing a more detailed understanding of the saturation levels between the hardware devices that manage data traffic. In addition, it provides another perspective on the relationship between frequency rates and the parameter configuration established in the processing of sample recordings.

By validating the hardware implementation against a software reference using quantitative similarity metrics, this work provides practical design guidelines for deploying SCNN layers on resource-constrained neuromorphic hardware. These results establish a foundation for the future integration of multi-layer SCNNs on FPGA platforms, enabling efficient, real-time, event-driven vision systems with predictable behavior and controlled resource usage.

## 5. Conclusions

This article presents a study of a convolutional system deployed on FPGA, with the aim of determining the values of the configurable parameters in its design that provide a stable performance for the convolution operation itself. To this end, the first part determined the input and output frequency limits of the SLIF neuron implemented on hardware, corroborating its behavior in accordance with the theoretical model of the LIF neuron. Subsequently, sample recordings from the MNIST-DVS dataset were used to compare convolution approaches.

The experimental design considered various combinations of values for the configuration parameters representative of the properties of the SLIF neuron, using only those that generated the largest number of output spikes. At this point, to contrast results, Sobel kernels were used on histograms of the sample recordings to determine vertical and horizontal edges.

The results obtained from the experiment, although still subject to improvement, confirm the viability of using the convolutional processor, characterized by limited memory and data transfer resources, as an accelerator for a layer of an SCNN model to be deployed in embedded hardware. In future work, the aim is to adapt it to process several convolutional layers for an SCNN model in the same processor.

## Figures and Tables

**Table 1:** Comparison of state-of-the-art DVS-based approaches and the proposed work.

Reference	Sensor	Task	Neural Model	Input Representation	Platform	Main Focus
Cordone et al. [16]	DVS	Gesture classification	SCNN	Voxel-based events	GPU	Sparse spiking convolution
Feng et al. [17]	DVS	Tracking, recognition	SNN	Raw events	Neuromorphic	Low-power spiking inference
Wan et al. [18]	DVS	Pedestrian detection	CNN	Event frames	CPU/GPU	Event-based detection efficiency
Baghaei Naeini et al. [19]	DVS	Force estimation	CRNN	Event stream	GPU	Temporal modeling of DVS data
Zheng and Zhou [21]	DVS	Object classification	SNN + attention	Event-driven convolution	Software	Learnable spiking attention
This work	DVS (MNIST-DVS)	Spiking convolution analysis	LIF-based SCNN	Raw events	FPGA (Zynq-7100)	Hardware-level characterization

**Table 2:** Quantitative comparison with related event-driven convolutional processors.

Work	Technology	Spiking Level	Max Kernel Size	Input/Output Rates	Throughput (Mop/s)	Energy Consumption (mW)
Linares-Barranco et al. (2010) [23]	FPGA, Spartan-3	I/O-core	11×11	–	24,6	–
Camuñas-Mesa et al. (2011) [24]	AMS035-CMOS	I/O-core	32×32	16/37 Mev/s	–	200
Camuñas-Mesa et al. (2018) [25]	FPGA, Spartan-6	I/O-core	32×32	10/10 Mev/s	∼3	0.85
Sommer et al. (2022) [28]	FPGA, ZynqU+ 7EV	core	3×3	33 FPS	-	2110
Zhang et al. (2022) [26]	FPGA, Zynq 7000	core	32×32	10 Mev/s	1,2	-
Aung et al. (2023) [30]	FPGA, KintexU+	core	3×3	543K FPS	-	53.000
Chen et al. (2024) [31]	FPGA, Virtex7-2000T	core	3×3	750 FPS	204.000	1500
Li et al. (2025) [32]	FPGA	core	3×3	680 FPS	-	4800
Ye et al. (2026) [33]	FPGA	core	3×3	153 FPS	183.000	1800
This work: Tapiador-Morales et al. (2019) [34]	FPGA (Zynq-7100)	I/O-core	7×7	0.77/9 Mev/s	348	0.92

**Table 3:** Main parameters used in Vivado for power estimation.

Parameter	Value (Units)
Ambient temperature	25 °C
Process	typical
Temp grade	extended
Toggle rate	12.5%
Vccint/Vccaux	1.0/1.8 V

**Table 4:** Configuration values for the model parameters.

LIF Parameters	Value
Threshold	80
Decay	10
Leakage value	80
Leakage prescaler	10
Kernel dimension	5
Refractory period value	50
Refractory period prescaler	8
Kernel type	Sobel

**Table 5:** Configuration values: six values were considered for the firing threshold (step size of 10), five values for leakage decay (step size of 5), five values for leakage value (step size of 20), and two values for refractory period value.

Group	Parameter	Values
1	Threshold	[50:10:100]
	Leakage decay	[0:5:20]
	Leakage value ($V_{lk}$)	[0:20:80]
	Ref. period value ($V_{rp}$)	[0:1]
2	Leakage prescaler ($P_{lk}$)	[0:1:20]
	Ref. period prescaler ($P_{rp}$)	[0:1:20]

**Table 6:** Loss of events after convolution.

Class	Input Th. (Kev/s)	$P_{\mathrm{lk}}=10,\;P_{\mathrm{rp}}=8$		$P_{\mathrm{lk}}=19,\;P_{\mathrm{rp}}=14$	
		Output Th. (Kev/s)	Loss (%)	Output Th. (Kev/s)	Loss (%)
0	42.42	22.94 ± 0.93	45.92	35.42 ± 1.68	16.51
1	26.84	14.26 ± 0.57	46.88	22.05 ± 0.62	17.85
2	36.03	18.78 ± 0.29	47.87	29.39 ± 0.35	18.41
3	27.98	13.14 ± 0.34	53.05	22.8 ± 0.25	18.52
4	28.56	13.46 ± 0.11	52.85	23.46 ± 0.29	17.83
5	18.98	8.74 ± 0.42	53.95	15.24 ± 0.65	19.72
6	29.73	15.3 ± 0.57	48.54	24.54 ± 1.08	17.46
7	18.09	8.56 ± 0.12	52.68	14.89 ± 0.18	17.67
8	32.65	17.07 ± 0.42	47.72	26.95 ± 1.03	17.46
9	35.76	19.28 ± 0.96	46.1	29.39 ± 1.25	17.82
AVG	29.70	15.15 ± 0.47	49.55	24.41 ± 0.74	17.93

**Table 7:** Metrics comparison results.

Sobel Kernel	Section	Metric	Value (min, max)
Vertical	H1	BCR	(0.9321, 0.9736)
		RMSE	(0.1755, 0.1971)
		Jensen–Shannon	(0.0284, 0.1018)
	H2	BCR	(0.9337, 0.9696)
		RMSE	(0.1751, 0.2028)
		Jensen–Shannon	(0.0253, 0.1295)
	H3	BCR	(0.9330, 0.9637)
		RMSE	(0.1749, 0.2259)
		Jensen–Shannon	(0.0359, 0.0996)
Horizontal	H1	BCR	(0.9283, 0.9324)
		RMSE	(0.1752, 0.1959)
		Jensen–Shannon	(0.1431, 0.3579)
	H2	BCR	(0.9331, 0.9578)
		RMSE	(0.1748, 0.2045)
		Jensen–Shannon	(0.1615, 0.3076)
	H3	BCR	(0.9354, 0.9698)
		RMSE	(0.1749, 0.2249)
		Jensen–Shannon	(0.0481, 0.3563)

## Data Availability

The original data presented in the study are openly available in http://imse-cnm.csic.es/caviar/MNIST_DVS accessed on 20 February 2026.
